# Study on the mechanism of inhibition of Escherichia coli by Polygonum capitatum based on network pharmacology and molecular docking technology: A review

**DOI:** 10.1097/MD.0000000000038536

**Published:** 2024-06-14

**Authors:** Shunhuan Chen, Dongyan Zhai, Yuan Li, Yong Tan, Xiaoke Tang, Xiang Pu, Yihui Chai, Lailai Li

**Affiliations:** a Guizhou University of Traditional Chinese Medicine, Guiyang, Guizhou, China.

**Keywords:** action mechanism, Escherichia coli, molecular docking, Polygonum capitatum

## Abstract

This study aims to analyze the effective components of Polygonum capitatum (PC) inhibiting Escherichia coli based on network pharmacology methods and predict its molecular mechanism of action. PC compounds and targets were collected from the TCMSP database, Swiss Target Prediction, and the literature. *E coli* targets were searched using the GeneCards database. The targets of *E coli* and the targets of the active ingredients of PC were taken as intersections to obtain the intersecting targets. The resulting overlapping targets were uploaded to the STRING database to construct the protein interaction network diagram of *E coli* target inhibition. The key targets for the inhibitory effect of PC on *E coli* were obtained. Gene ontology and Kyoto Encyclopedia of Genes and Genomes pathway enrichment analyses were performed by uploading key targets into the DAVID database. The results showed that there were 50 targets for PC to inhibit *E coli*. Among them, there are 5 core targets, mainly including AKT1, TNF, EGFR, JUN, and ESR1. A total of 196 gene ontology functional analysis results and 126 Kyoto Encyclopedia of Genes and Genomes pathway enrichment analysis results were obtained. These include cellular response to cadmium-ion, cellular response to reactive oxygen species, pathways in cancer, prostate cancer, and PI3K-Akt signaling pathway. Molecular docking results indicate that Lutedin, Hirsutin, Flazin, and Ellagic acid in PC have high affinity for the target genes AKT1, TNF, MAPK3 and EGFR. PC exerts its inhibitory effect on *E coli* through multi-targets and multi-pathways, which provides a new basis for the new use of PC as an old medicine.

## 1. Introduction

Escherichia coli is a gram-negative opportunistic pathogen that is widely distributed in the intestines of animals and nature. It has long been considered part of the normal gut microbiota. It is considered a nonpathogenic bacterium. Some specific *E coli* serotypes were pathogenic to humans and animals until the middle of the 20th century. Based on different biological characteristics, pathogenic *E coli* is divided into 6 categories: enteropathic *E coli*, enterotoxic *E coli*, enteroinvasive *E coli*, enterohemorrhagic *E coli*, enteric adhering *E coli* and diffuse adhering *E coli. E coli* is an increasingly common cause of serious bacterial diseases in humans and animals. Sepsis, pyelonephritis and peritonitis are commonly caused by *E coli* infection.^[1]^ *E coli* infections are particularly common in the urinary tract. During the infection process, pattern recognition receptors expressed by immune cells can recognize *E coli*, activate downstream inflammatory signaling pathways, mediate cytokine secretion and regulate inflammatory responses. When *E coli* invades the animal body, host immune cells such as macrophages and dendritic cells can recognize pattern recognition receptors through the cell membrane or intracellular structure, including Toll-like receptors and Nod-like receptors, identify its specific pathogen-associated molecular pattern or damage-associated molecular pattern, and then activate intracellular signaling pathways to induce the secretion of cytokines and chemokines, thereby exerting an anti-infection effect.^[2]^ Most strains in the *E coli* group are harmful, with only 1% of *E coli* being probiotic in the gut and the rest being pathogenic bacteria. The toxins they produce attach to host cells, interfere with cell metabolism and disrupt cell tissue structure. Thousands of people are infected with *E coli* and many die without treatment. *E coli* often contaminates unsterilized milk, fruit juices, undercooked meat products, fresh fruit and vegetables. In recent years, there has been an explosion in cases of *E coli* contamination of medical systems and agricultural products. *E coli* originates from feces and causes cross-contamination of food and water through fecal contamination, leading to infection directly from human or animal consumption. Typically, people who consume untreated meat products, raw milk, and fresh vegetables and fruit are susceptible to infection and illness caused by *E coli*. When *E coli* multiplies to a certain number (10^6^–10^9^), it causes the host to develop characteristics of disease. Asymptomatic hosts and carriers are both carriers and sources of *E coli* infection. Research has shown that *E coli* and Klebsiella pneumoniae are susceptible to various antibiotics, with higher susceptibility to imipenem, meropenem and tigecycline.^[3]^ *E coli* tends to develop drug resistance and should not be treated with antibiotics for long periods of time. Traditional Chinese Medicine has a long medicinal history and rich clinical experience. Previous literature has shown that Traditional Chinese Medicine has antibacterial and immunoregulatory effects and is an effective treatment for urinary tract infections caused by clinically resistant bacteria. Therefore, it is necessary to investigate the inhibitory effect of Traditional Chinese Medicine Polygonum capitatum (PC) on *E coli*.

PC, also known as Sijihong, Shimangcao in China, is a perennial herb of PC group in Polygonaceae, mainly distributed in Guizhou, Yunnan, Sichuan, Xizang, Guangxi and other provinces and regions.^[4]^ It is one of the commonly used folk Miao medicines in China.^[5]^ Clinical studies have found that it has significant therapeutic effects on multiple systemic diseases such as the lower urinary tract. Modern pharmacological studies have found that it has anti-inflammatory, antibacterial, antioxidant, and hypoglycemic effects.^[6]^ PC is a genuine medicinal herb in Guizhou Province, mainly used in clinical practice to treat urinary tract infections. Urinary system infection is one of the common infectious diseases that is only inferior to respiratory tract infection in clinic,^[7]^ the main pathogenic bacteria are *E coli*, followed by Proteobacterium and *K pneumoniae*.^[8]^ Shang-Gao Liao et al demonstrated through experiments that 7 different polar parts of the 70% ethanol extract of PC have high antibacterial activity against *E coli*, with a minimum inhibitory concentration of 0.20 mg/mL and a minimum bactericidal concentration of 0.78 mg/mL.^[9]^ Zhang C et al identified 4 highly polar parts out of the 7 effective parts of PC alcohol extract as their main antibacterial components, namely 6-gallic acid acyl glucose, 3,6-diglycidyl glucose, 1,3,6-triglycidyl glucose, and Davidiin.^[10]^ Ren G et al experimentally determined that the water extract of PC can resist the infection of *E coli* and reduce the mortality rate of mice infected with *E coli*.^[11]^

Therefore, this study focuses on studying the potential mechanism of inhibitory effect of PC on *E coli*, laying the foundation for future research on the antibacterial activity of PC.

## 2. Materials and methods

**Figure 1.**
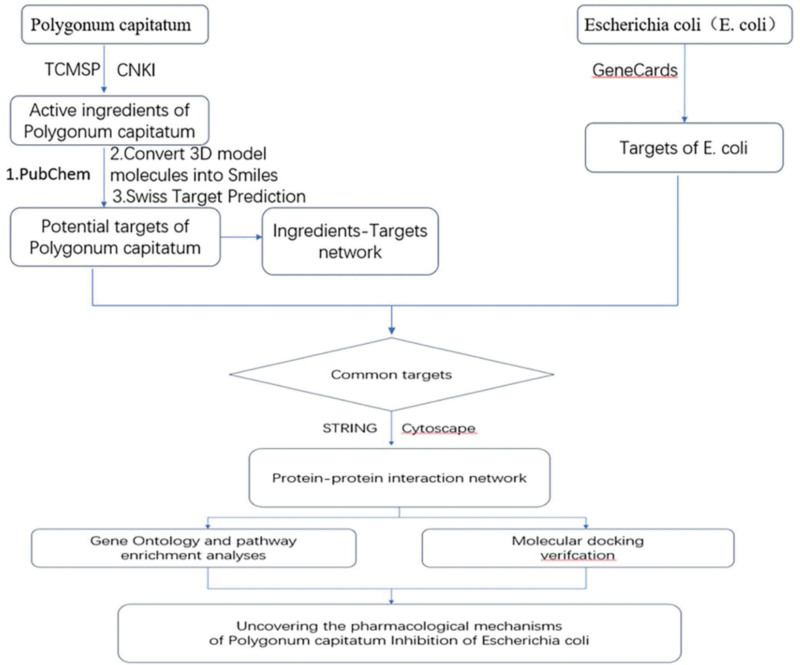
Flowchart of pharmacological research on the use of Polygonum capitatum for inhibiting Escherichia coli.

The flow chart of the study design is shown in Figure 1.

### 2.1. Collection of active ingredients and potential targets of PC

The main active components of PC were obtained through literature search on CNKI using the keywords “Polygonum capitatum” and “Chemical components of Polygonum capitatum”. Screening of chemical components that have been entered from TCMSP. Oral bioavailability is an important parameter of drug absorption and metabolism. The higher the value, the better the biological activity and drug-like drug-like properties (DL) of the drug. Therefore, a standard oral bioavailability ≥ 30% and a DL ≥ 0.18 are commonly used in the literature as the selection criteria for active ingredient screening. Then, the Smiles were retrieved on the Swiss Target Prediction (http://www.Swisstargetprediction.ch) and targets with *P* > .1 were selected. The predicted targets of the final active ingredients were obtained.

### 2.2. Screening of Bacillus coli targets and construction of active ingredient-target network diagram

The screening of active ingredient targets related to the action of *E coli* in PC were achieved by searching for *E coli*-related target proteins using the keyword Bacillus coli in the GeneCards database (https://www.genecards.org/), removing duplicate genes, and conducting matching analysis with the active ingredient targets of PC. The core targets are obtained by extracting common genes between the component targets of PC and the targets of *E coli*. Then, a “component-target” network is constructed by importing the screened active ingredients and their core targets into Cytoscape 3.9.1 software.

### 2.3. Construction of protein-protein interaction network

The construction of a target protein interaction (PPI) network is achieved by importing the screened potential targets into the STRING database (https://string-db.org/cgi/input?sessionId=bR2DXqok3ofm) and setting the species to “Homo sapiens,” where lines represent the relationship between target protein interactions. The final PPI network diagram is obtained by importing the tsv file of the analysis results into Cytoscape 3.9.1 software for visualization. The core targets are the top 10 nodes with the highest degree value that were finally filtered out.

### 2.4. Gene ontology and Kyoto Encyclopedia of Genes and Genomes pathway analyses

The gene ontology (GO) and Kyoto Encyclopedia of Genes and Genomes (KEGG) pathway analyses were the final analysis results obtained using the DAVID 6.8 database.

### 2.5. Construction of a network diagram of active ingredients–disease targets–signaling pathways

The component target pathway network is constructed by importing active ingredients, target genes, and enrichment pathways into Cytoscape 3.9.1 software, and finally conducting topological parameter analysis.

### 2.6. Molecular docking verification

The structure of the drug small molecule (Mol2 format) was downloaded from the TCMSP database. The 3D structure of the target protein (PDB format) was downloaded from PDB Database (https://www.rcsb.org/). Then dehydrated the ligand by PyMOL software (Schrödinger). The target protein was hydrogenated and converted to pdbqt format by AutoDock software, and the drug small molecule rotation bond was set up and saved in pdbqt format. The location of the active pocket built depends on the original ligand’s position. The X, Y, and Z centers were adjusted on the original ligand of different receptors. Receptor proteins and ligand small molecules are obtained by selecting the top 5 key targets in the degree ranking of the PPI network and the core components in the top 5 moderate values of “Traditional Chinese Medicine - Active Ingredients - Target.” When the drug molecular ligand binds to the target to form conformational stability, the lower the energy is, the more stable the structure is. The molecular docking results are visualized using Pymol.

## 3. Result

### 3.1. The chemical components and gene targets of PC

Forty-five main chemical components of PC were obtained from the TCMSP database. Oral bioavailability ≥ 30% and DL ≥ 0.18 were used as screening conditions for the active components of PC. A total of 11 active components were obtained, among which (+)-Catechin had no corresponding target or disease. Therefore, there are 10 active ingredient targets. Then, TCMSP was combined with PubChem (https://pubchem.ncbi.nlm.nih.gov/) and 3D model molecules to convert them into Smiles to obtain the Smiles number. The targets with *P* > .1 were selected by Smiles searching on the Swiss Target Prediction. After screening, a total of 225 predicted targets for active ingredients were obtained. After sorting and screening, there are 8 active ingredients available as shown in Table 1.

**Table 1:** Active ingredients in Polygonum capitatum.

MolId	MolName	OB (%)	DL
MOL000006	Luteolin	36.16	0.25
MOL000098	Quercetin	46.43	0.28
MOL001002	Ellagic acid	43.06	0.43
MOL005073	Ethyl brevifolincarboxylate	30.86	0.33
MOL000569	Digallate	61.85	0.26
MOL001001	Quercetin-3-o-*β*-d-Glucuronide	30.66	0.74
MOL009295	Flazin	94.28	0.39
MOL008487	Hirsutine	34.44	0.43

OB = oral bioavailability, DL = drug-like properties.

### 3.2. Active constituents of PC–targets network diagram

**Figure 2.**
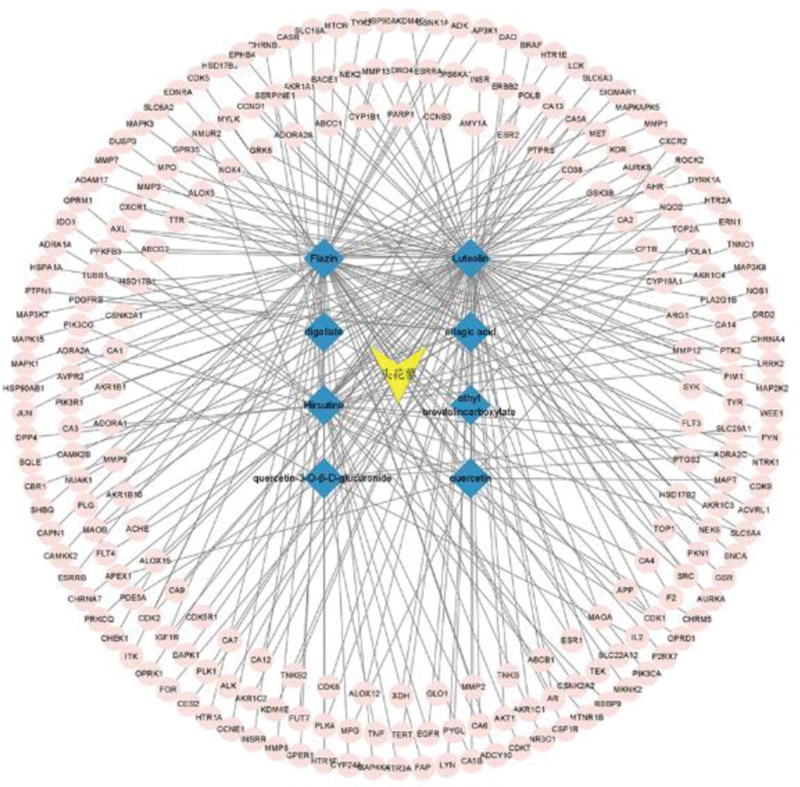
Polygonum capitatum–active ingredients–target network.

The 225 target proteins predicted by TCMSP were converted into corresponding genes by using the UniProt database (https://www.uniprot.org/) with the species set as humans. The construction of drug active ingredient-target network diagram is achieved by importing the selected 8 active ingredients and their targets into Cytoscape 3.9.1 software, as shown in Figure 2. The network contains a total of 234 nodes and 270 lines. This study also used the plugin CytoNCA in Cytoscape 3.9.1 software to analyze each node in the network graph and obtain the degree values of each node in the network. Among all active ingredient nodes, the top 5 with the highest degree values are Luteolin, Flazin, Hirsutine, Ellagic acid, and Quercetin-3-o-*β*-d-glucuronide acid. These components are the main active ingredients of PC that inhibit *E coli*. The degree represents the number of targets corresponding to the active ingredient in the active ingredient node, and the number of active ingredients that can interact with the target in the target node. This means that the larger the degree value, the greater the role it plays in the inhibition of *E coli* by PC.

### 3.3. Screening of potential targets for inhibition of Bacillus coli by PC

**Figure 3.**
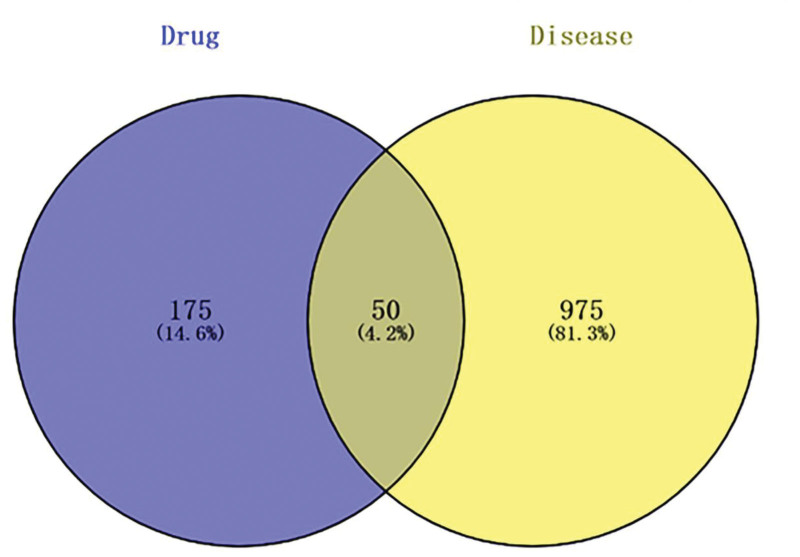
Polygonum capitatum–*E coli* Wayne diagram. *E coli* = Escherichia coli.

A total of 1025 *E coli* genes were found by using the keyword “Bacillus coli” in the GeneCards database. Fifty common target genes were obtained by importing 225 target genes of PC and 1025 Bacillus coli-related genes mined from the GeneCards database into the Venny 2.1.0 online tool (https://bioinfogp.cnb.csic.es/tools/venny/), which are potential targets for PC to inhibit Bacillus coli. See Figure 3.

### 3.4. Target PPI results

**Figure 4.**
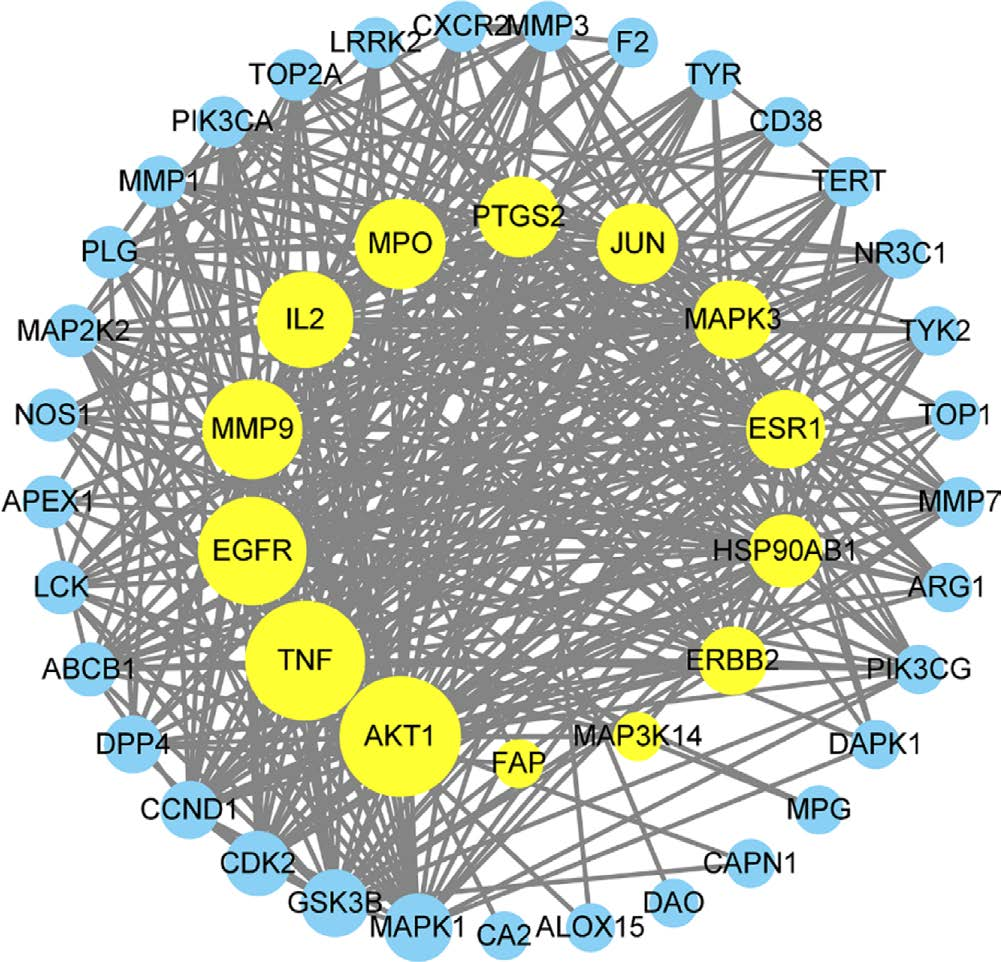
PPI network. PPI = protein-protein interaction.

**Figure 5.**
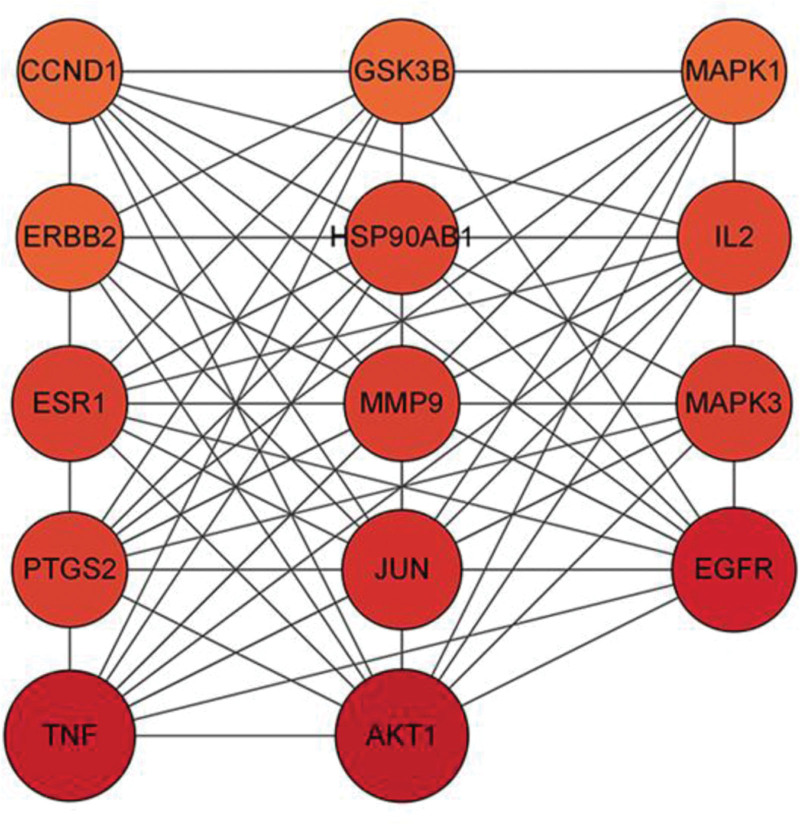
Core targets interaction network.

The PPI relationship network diagram was obtained by uploading the intersection of the main active ingredients screened from PC and the targets of PC inhibiting *E coli* to the STRING database. The PPI network presents a total of 47 active nodes and 363 interaction lines in the image. The result obtained is to import the data into Cytoscape 3.9.1 for processing, as shown in Figure 4. The first 14 nodes are selected based on the degree value, including AKT1, TNF, EGFR, JUN, ESR1, MMP9, PTGS2, MAPK3, IL2, HSP90AB1, etc. These 14 targets are the core targets for the inhibitory effect of PC on *E coli*, as shown in Figure 5.

### 3.5. GO and KEGG pathway analyses

The 50 key targets for the anti-*E coli* activity of PC selected were analyzed using the DAVID platform for GO and KEGG analysis.

**Figure 6.**
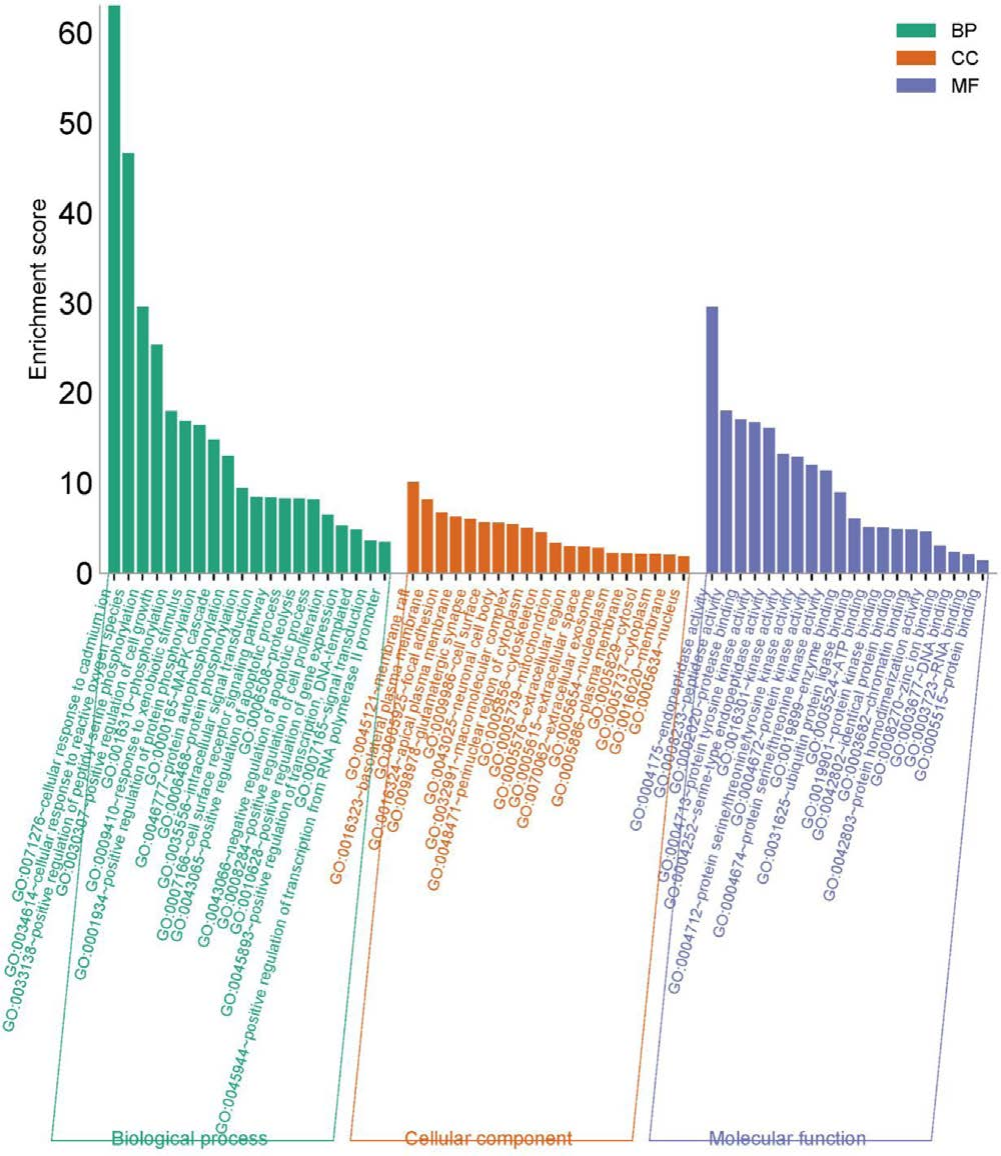
GO enrichment analysis. GO = gene ontology.

GO analysis selects the top 20 entries of biological processes (BPs), cellular components, and molecular functions. The GO enrichment analysis results indicate that the BP analysis has a significant impact on cellular response to cadmium-ion, reactive oxygen specifications, positive regulation of peptide series phonology, positive regulation of cell growth, phonology, response to xenobiotic stimuli, etc. The significant effects of cellular component analysis enrichment include membrane raft, basolateral plasma membrane, focal adhesion, apical plasma membrane, glutamatergic synapse, cell surface, etc. The most significant effects of molecular function analysis enrichment are endpoint activity, peptide activity, protein binding, protein tyrosine kinase activity, serine type endpoint activity, kinase activity, etc as shown in Figure 6.

**Figure 7.**
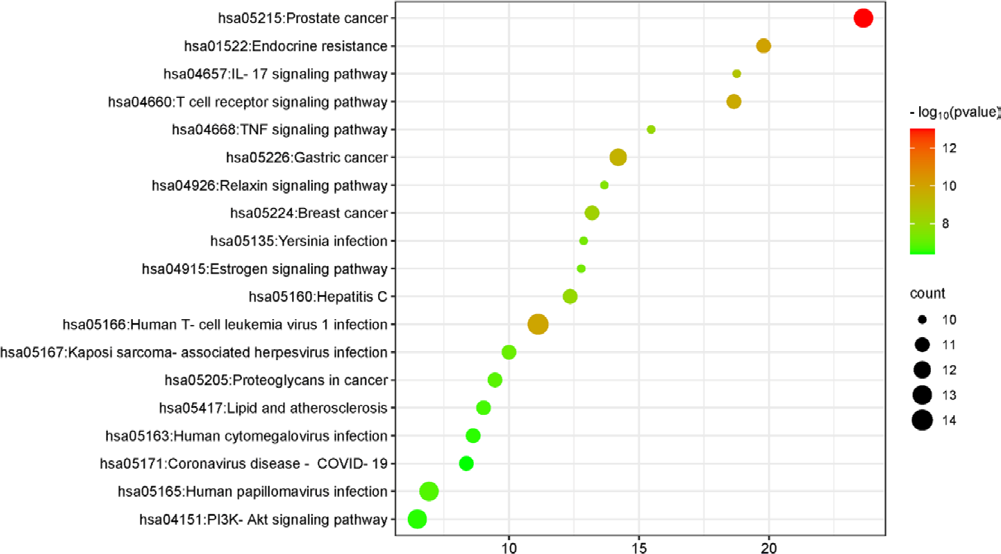
KEGG enrichment analysis. KEGG = Kyoto Encyclopedia of Genes and Genomes.

The KEGG pathway analysis results indicate that the inhibition of *E coli* by PC is closely related to multiple signaling pathways such as pathways in cancer, human T-cell leukemia virus 1 infection, prostate cancer, human papillomavirus infection, PI3K-Akt signaling pathway, and gastric cancer, revealing that PC may inhibit the process of *E coli* by targeting these pathways, as shown in Figure 7.

### 3.6. Construction of a network diagram of active ingredients–disease targets–signaling pathways

**Figure 8.**
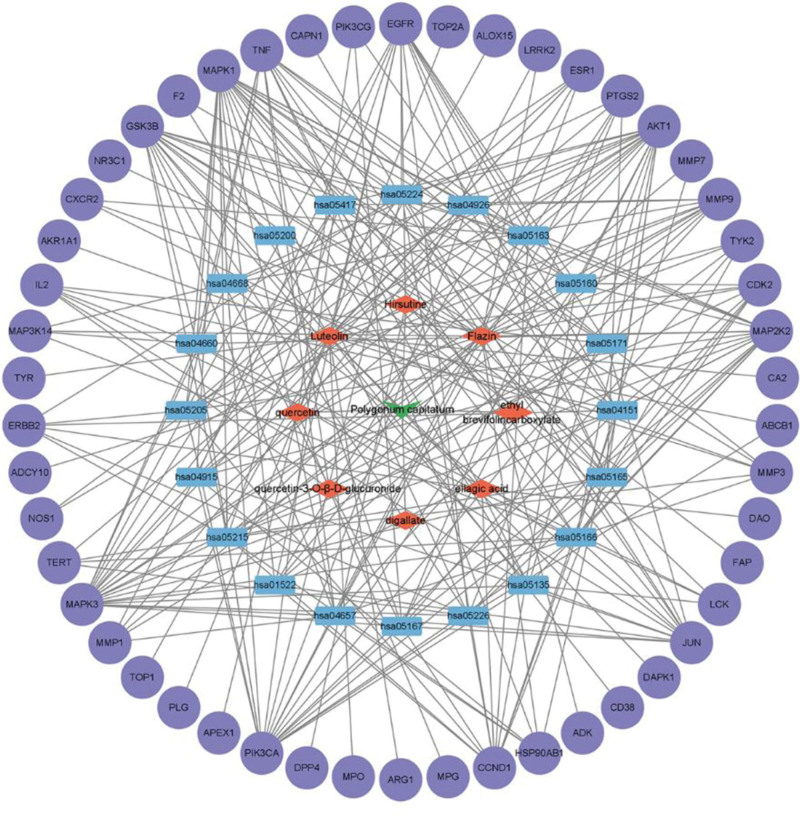
Active ingredient–disease target–signaling pathway network diagram.

The construction of the “active ingredient - disease target - signal pathway” network diagram was made by importing 8 screened active ingredients, 50 target genes, and 20 *E coli*-related pathways into Cytoscape 3.7.1 software, as shown in Figure 8. The top 5 compounds with degree values are Luteolin, Flazin, Hirsutine, ellagic acid, and Quercetin-3-o-*β*-d-glucuronide. It can be inferred that the above 5 compounds are potential active ingredients of PC for inhibiting *E coli*.

### 3.7. Molecular docking results

**Figure 9.**
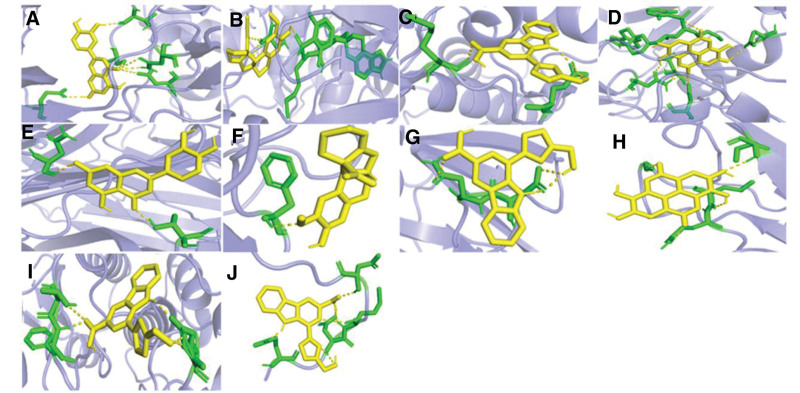
Visualization of molecular docking between active ingredients and core targets.

**Figure 10.**
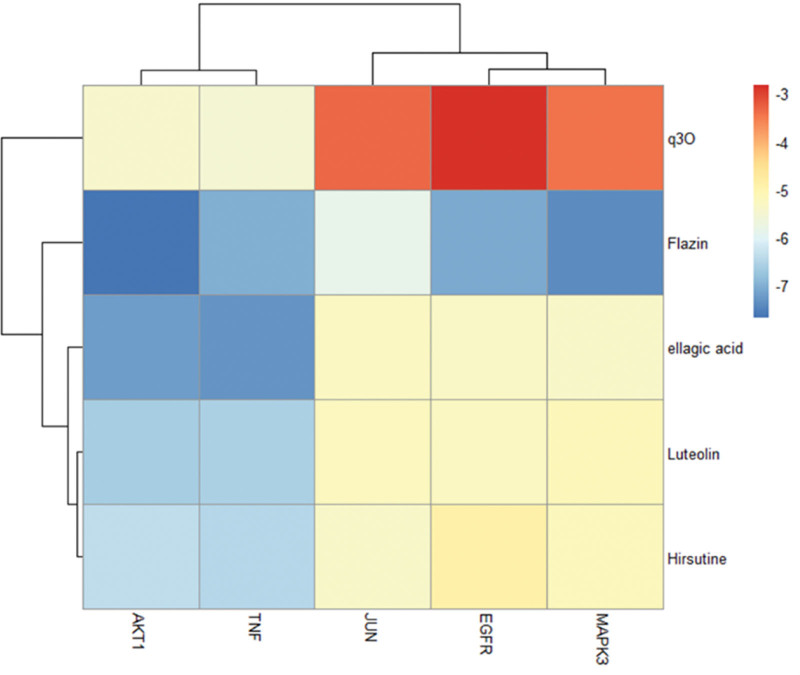
Molecular docking result heat map.

According to Table 2, the top 4 targets of degree are AKT1, TNF, MAPK3, and EGFR. AKT1 has strong binding activity with Lutein, Hirsutine, Flazin, Ellagic acid, TNF with Lutein, Hirsutine, Flazin, ellagic acid, MAPK3 with Flazin, EGFR with Flazin. Their binding energy is all <−5.0 kcal·mol^−1^, showing good binding force. The binding of them are shown in Figure 9. Figure 10 shows the heat map of molecular docking results, with darker blue indicating better binding effect.

**Table 2:** Molecular docking binding energy.

MolId	Active	Binding energy (Kcal/mol)				
		AKT1	TNF	EGFR	JUN	MAPK3
MOL000006	Luteolin	−6.65	−6.57	−5.27	−5.25	−5.12
MOL001001	Quercetin-3-o-*β*-d-glucuronide	−5.43	−5.54	−2.83	−3.34	−3.44
MOL008487	Hirsutine	−6.40	−6.50	−4.88	−5.36	−5.16
MOL009295	Flazin	−7.68	−7.01	−7.05	−5.86	−7.39
MOL001002	Ellagic acid	−7.21	−7.31	−5.32	−5.30	−5.40

## 4. Discussion

*E coli* infection usually causes sepsis, pyelonephritis, and peritonitis,^[1]^ particularly prone to ascending infections and causing urinary tract infections. Untreated urinary tract infections can lead to acute and chronic pyelonephritis, scarring of the kidney and even kidney failure.^[12–15]^ Carly Hennessey and others have shown that *E coli* infection significantly reduces the diversity of the bacterial flora in the cecum, which may disrupt microbiota-mucosal interactions, affecting both local and systemic immune responses, and thus exacerbating the infection.^[16]^

Antibiotics are the primary treatment for bacterial infections.^[17]^ Bacterial resistance increases over time, reducing the effectiveness of antibiotics.^[18]^ In addition, antibiotics can cause toxic and allergic reactions, and double infections with prolonged use. However, traditional Chinese herbs have precise clinical efficacy and a long history of medicinal use.^[19]^ Clinical studies have shown that the efficacy of a single-party preparation of the PC is reliable in the treatment of urinary tract infections. However, since the active components of PC that inhibit *E coli* and its mechanism of action are still unclear, the present study explored the potential mechanism of PC’s action on *E coli* through network pharmacological analyses.

PPI network analysis revealed that the core targets of PC in inhibiting *E coli* were AKT1, TNF, EGFR, JUN, and MAPK3. AKT1 plays a central role in regulating the cellular processes of bacteria. LIU G et al found that AKT1 is downregulated during bacterial infection. In mouse model of acute inflammatory lung injury and Staphylococcus aureus infection, AKT1 deficiency leads to severe disease progression accompanied by neutrophil recruitment and enhanced bactericidal activity.^[20]^ TNF is a multifunctional pro-inflammatory signaling factor, which binds to specific receptors on cell membranes and participates in the activation, function and differentiation of immunomodulatory cells, and has the ability to regulate the prevention of malignant bacterial infections, the immune system, and cell apoptosis.^[21]^ TNF can participate in the immune response by activating T-cell immunoreactivity and has the ability to kill transformed cells and certain virus-infected cells. TNF-α is an inflammatory cytokine secreted by mast, endothelial, and macrophage cells that possesses a variety of biological activities. TNF-α can transmit biological information to the nucleus of the cell through specific receptors present on the cell membrane to produce cytotoxicity, immunomodulation, antitumor, antiviral, inflammation-mediated, and other biological activities.^[22]^ EGFR is a transmembrane protein whose expression plays an important role in initiating inflammatory factors.^[23]^ EGFR can sometimes inhibit bacterial infections.^[24]^ In summary, proteins such as AKT1, TNF and EGFR play important roles in the body’s antimicrobial, anti-infective, inflammatory and immune processes. Network pharmacological analyses suggest that PC can act synergistically by regulating the above proteins to exert anti-*E coli*-induced infections.

Most of the inhibitory processes are associated with inflammatory responses as well as the development of cancer. Research has shown that cancer patients are more susceptible to a variety of bacterial infections, particularly urinary tract infections caused by urinary pathogenic *E coli*.^[25]^ Certain strains of *E coli* in the gut can influence the onset and development of colorectal cancer (CRC) by using virulence factors and inflammatory pathways.^[26]^ Many studies have shown that chronic inflammation induced by microorganisms (including *E coli*) during inflammatory bowel disease (IBD) makes individuals more susceptible to CRC. Evidence supporting the role of *E coli* in the etiology of CRC through IBD, not limited to chronic inflammation. During IBD and CRC, the growth of *E coli* as an intracellular pathogen enables the bacteria to regulate the host cell cycle, induce DNA damage, and accumulate mutations, these are some of the contributing factors behind the etiology of colon cancer.^[27]^ The PI3K-Akt pathway mediates cell growth, differentiation, proliferation, and other processes.^[28]^ It was reported that a variety of pathogenic bacteria can enhance bacterial invasion and promote cellular internalization through the activation of the PI3K-Akt pathway, e.g. Listeria monocytogenes, *E coli* K1 strain, Pseudomonas aeruginosa, Streptococcus pneumoniae and *K pneumoniae*.^[29]^ The PI3K-Akt pathway can also regulate nuclear factors κB inhibits the binding of proteases, activates autophagy response, and thus participates in inflammatory response.^[30]^ TNF pathway is involved in various cancers and immune-mediated diseases by activating nuclear factor κB to regulate cell proliferation, apoptosis, differentiation, and mediate BPs such as antiviral, antimicrobial, immunomodulatory, and apoptotic processes.^[31]^ The results of GO enrichment analysis and KEGG analysis suggested that the cancer pathway, PI3K-Akt pathway, and TNF pathway were the key pathways in the inhibition of *E coli* by PC.

In this paper, network pharmacological analyses showed that PC exerted its inhibitory effect on *E coli* mainly through Luteolin, Quercetin, Ellagic acid, Ethyl brevifolincarboxylate, Digallate, and Quercetin-3-o- *β*-d-glucuronide, Flazin, Hirsutine to modulate multiple targets. Luteolin have significant efficacy in anti-inflammation.^[32,33]^ It modulates multiple inflammatory mediators and alters various signaling pathways involved in inflammation. Luteolin inhibits NF-κB activity and the expression of inflammatory factors such as TNF-α.^[34]^ Luteolin may exert anticancer activity by downregulating key regulatory pathways associated with tumorigenesis, inducing oxidative stress, cell cycle arrest, upregulating apoptotic genes, and inhibiting cell proliferation and angiogenesis in cancer cells.^[35]^ Existing studies have shown that Quercetin has a wide range of physiological activities such as antibacterial,^[36]^ antiviral,^[37]^ anti-inflammatory,^[38]^ and antioxidant.^[39]^ Hong Z et al found that Quercetin could inhibit a variety of bacteria, such as Staphylococcus aureus and *E coli*. Among them the inhibition of Quercetin on *E coli* via disrupting the structure of the bacterial cell wall, altering the expression profile of bacterial proteins.^[40]^ Hirsutine has pharmacological effects such as anti-inflammatory and anticancer.^[41]^ Wang S et al showed that Hirsutine inhibited the proliferation, migration, and invasion of colon cancer cells and promoted their apoptosis.^[42]^ The above studies have shown that compounds such as Luteolin, Quercetin, and Hirsutine are the key compounds in the inhibitory effect of PC on *E coli*. The mechanism involves multiple signal pathways, through multiple pathways and targets, which are mainly related to inflammatory response, cancer pathway, and TNF signaling pathway, mediated by PI3K-Akt signaling pathway. This study provides a theoretical basis for the mechanism of PC in inhibiting *E coli* infection in the future.

## 5. Conclusion

In this study, we used network pharmacology and molecular docking approaches to predict the potential mechanism of *E coli* inhibition by PC. The results showed that Luteolin, Quercetin and Hirsutine were the major active compounds of PC. Meanwhile, AKT1, TNF and EGFR targets are potential targets for the inhibition of *E coli* by PC. PC inhibits the development of *E coli* by modulating the inflammatory response, the cancer pathway, the TNF signaling pathway and the PI3K-Akt signaling pathway. This study provides a reference for further research on the molecular mechanism of PC against *E coli*.

### 5.1. Strengths and limitations of this study

However, this study has some limitations. First, the TCMSP was used to identify the compounds in PC. Still, probably not all chemical compounds with biological activity were included, and compounds with unproven physical activity were excluded. Thus, the accuracy and timeliness of the database need to be further verified. Second, we identified the essential active compounds of PC through a network pharmacological approach. Still, these compounds only do not account for the complete mechanism and pathway of action of PC in inhabiting *E coli* and only serve as supporting evidence for the efficacy of PC. Finally, the effects of target proteins identified through the pharmacology network approach in the ponderous and sophisticated human system have not been fully explored and explained.

## Acknowledgments

We are very grateful to the TCMSP databases Genecard Database and PDB Database for providing information on the study and to all colleagues who participated in the study.

## Author contributions

**Conceptualization:** Xiang Pu, Lailai Li.

**Data curation:** Shunhuan Chen, Dongyan Zhai, Yuan Li, Yong Tan, Xiaoke Tang.

**Formal analysis:** Lailai Li.

**Funding acquisition:** Xiang Pu, Lailai Li.

**Investigation:** Shunhuan Chen.

**Methodology:** Shunhuan Chen, Yuan Li.

**Project administration:** Shunhuan Chen.

**Resources:** Shunhuan Chen.

**Software:** Shunhuan Chen, Dongyan Zhai, Yong Tan.

**Supervision:** Shunhuan Chen.

**Validation:** Shunhuan Chen.

**Visualization:** Shunhuan Chen, Xiaoke Tang.

**Writing – original draft:** Shunhuan Chen.

**Writing – review & editing:** Yihui Chai, Lailai Li.

## References

[1] Li CH Wang JH Redmond HP. Bacterial lipoprotein-induced self-tolerance and cross-tolerance to LPS are associated with reduced IRAK-1 expression and MyD88-IRAK complex formation. J Leukoc Biol. 2006;79:867–75. 16461741 10.1189/jlb.0905505

[2] Kawai T Akira S. The role of pattern-recognition receptors in innate immunity: update on Toll-like receptors. Nat Immunol. 2010;11:373–84. 20404851 10.1038/ni.1863

[3] M Hassan M Lagha R Mabrouk I. Molecular investigation of multidrug-resistant Escherichia coli clinical isolates from patients with urinary tract infections. Pak J Biol Sci. 2021;24:636–45. 34486339 10.3923/pjbs.2021.636.645

[4] Lin Y He L Chen X-J. Polygonum capitatum, the hmong medicinal flora: a comprehensive review of its P-phytochemical, pharmacological and pharmacokinetic characteristics. Molecules. 2022;27:6407. 36234943 10.3390/molecules27196407 PMC9571880

[5] Fan S Huang Y Zuo X. Exploring the molecular mechanism of action of Polygonum capitatum Buch-Ham. ex D. Don for the treatment of bacterial prostatitis based on network pharmacology and experimental verification. J Ethnopharmacol. 2022;291:115007. 35150815 10.1016/j.jep.2022.115007

[6] Chen H Yuan L Ma X. Herb-drug interaction: the effect of Polygonum capitatum extract on pharmacokinetics of levofloxacin in rats. J Pharm Biomed Anal. 2021;195:113832. 33349475 10.1016/j.jpba.2020.113832

[7] Geerlings SE. Clinical presentations and epidemiology of urinary tract infections. Microbiol Spectr. 2016;4:1. 10.1128/microbiolspec.UTI-0002-2012 27780014

[8] Ronald A. The etiology of urinary tract infection: traditional and emerging pathogens. Dis Mon. 2003;49:71–82. 12601338 10.1067/mda.2003.8

[9] Liao SG Zhang LJ Sun F. Antibacterial and anti-inflammatory effects of extracts and fractions from Polygonum capitatum. J Ethnopharmacol. 2011;134:1006–9. 21296143 10.1016/j.jep.2011.01.050

[10] Zhang C Zhang X Zhang Y Xu Q Xiao H Liang X. Analysis of estrogenic compounds in Polygonum cuspidatum by bioassay and high performance liquid chromatography. J Ethnopharmacol. 2006;105:223–8. 16377110 10.1016/j.jep.2005.10.029

[11] Ren G Chang F Lu S Zhong H Zhang G. Pharmacological studies of Polygonum capitatum Buch-Ham. ex D. Don. Zhongguo Zhong Yao Za Zhi. 1995;20:107–9, 128. 7779271

[12] Larcombe J. Urinary tract infection in children. BMJ. 1999;319:1173–5. 10541510 10.1136/bmj.319.7218.1173 PMC1116958

[13] Ladhani S Gransden W. Increasing antibiotic resistance among urinary tract isolates. Arch Dis Child. 2003;88:444–5. 12716722 10.1136/adc.88.5.444 PMC1719551

[14] Mir S Erdoğan H Güler S Şengül G Koyu A Aydemir S. Antibiotic resistance in pediatric urinary tract infection in the Egean Region. Ege Tip Dergisi. 2002;41:207–10.

[15] Wu CY Chiu PC Hsieh KS Chiu CL Shih CH Chiou YH. Childhood urinary tract infection: a clinical analysis of 597 cases. Acta Paediatr Taiwan. 2004;45:328–33. 15868848

[16] Hennessey C Keogh CE Barboza M. Neonatal enteropathogenic Escherichia coli infection disrupts microbiota-gut-brain axis signaling. Infect Immun. 2021;89:e0005921. 33820817 10.1128/IAI.00059-21 PMC8370682

[17] Singh SB Young K Silver LL. What is an “ideal” antibiotic? discovery challenges and path forward. Biochem Pharmacol. 2017;133:63–73. 28087253 10.1016/j.bcp.2017.01.003

[18] Shen X Zhang W Peng C. In vitro anti-bacterial activity and network pharmacology analysis of Sanguisorba officinalis L. against Helicobacter pylori infection. Chin Med. 2021;16:33. 33865425 10.1186/s13020-021-00442-1 PMC8052767

[19] Zhang L Bao M Liu B Zhao H Lu C. Effect of andrographolide and its analogs on bacterial infection: a review. Pharmacology. 2019;105:1–12. 31694037 10.1159/000503410

[20] Liu G Bi Y Wang R. Kinase AKT1 negatively controls neutrophil recruitment and function in mice. J Immunol. 2013;191:2680–90. 23904165 10.4049/jimmunol.1300736

[21] Mutai P Pavadai E Wiid I Ngwane A Baker B Chibale K. Synthesis, antimycobacterial evaluation and pharmacophore modeling of analogues of the natural product formononetin. Bioorg Med Chem Lett. 2015;25:2510–3. 25977095 10.1016/j.bmcl.2015.04.064

[22] Guan J Wang Z Liu X. IL-6 and IL-10 closely correlate with bacterial bloodstream infection. Iran J Immunol. 2020;17:185–203. 32996896 10.22034/iji.2020.87266.1793

[23] da Cunha Santos G Shepherd FA Tsao MS. EGFR mutations and lung cancer. Annu Rev Pathol. 2011;6:49–69. 20887192 10.1146/annurev-pathol-011110-130206

[24] Hardbower D Singh K Asim M. EGFR regulates macrophage activation and function in bacterial infection. J Clin Invest. 2016;126:3296–312. 27482886 10.1172/JCI83585 PMC5004944

[25] Mahmoud AT Salim MT Ibrahem RA Gabr A Halby HM. Multiple drug resistance patterns in various phylogenetic groups of hospital-acquired uropathogenic E. coli isolated from cancer patients. Antibiotics (Basel). 2020;9:108. 32131426 10.3390/antibiotics9030108 PMC7148488

[26] Nouri R Hasani A Shirazi KM. Escherichia coli and colorectal cancer: unfolding the enigmatic relationship. Curr Pharm Biotechnol. 2022;23:1257–68. 34514986 10.2174/1389201022666210910094827

[27] Khan AA Khan Z Malik A. Colorectal cancer-inflammatory bowel disease nexus and felony of Escherichia coli. Life Sci. 2017;180:60–7. 28506682 10.1016/j.lfs.2017.05.016

[28] Song Z Guo Y Zhou M Zhang X. The PI3K/p-Akt signaling pathway participates in calcitriol ameliorating podocyte injury in DN rats. Metabolism. 2014;63:1324–33. 25044177 10.1016/j.metabol.2014.06.013

[29] Zhou J Gao Y LIu J Tang X Liang D Chen L. Molecular mechanism of Chuanxinlian against bacteria based on network pharmacology. Pharm Clin Chin Materia Medica. 2021;12:22–6.

[30] Liu R Chen Y Liu G. PI3K/AKT pathway as a key link modulates the multidrug resistance of cancers. Cell Death Dis. 2020;11:797. 32973135 10.1038/s41419-020-02998-6 PMC7515865

[31] Montfort A Colacios C Levade T Andrieu-Abadie N Meyer N Ségui B. The TNF paradox in cancer progression and immunotherapy. Front Immunol. 2019;10:1818. 31417576 10.3389/fimmu.2019.01818 PMC6685295

[32] Fan X Du K Li N. Evaluation of anti-nociceptive and anti-inflammatory effect of Luteolin in mice. J Environ Pathol Toxicol Oncol. 2018;37:351–64. 30806241 10.1615/JEnvironPatholToxicolOncol.2018027666

[33] Hu Z Tong L Geng Y Yang Q Hou J. A review on pharmacological activities and preparations of luteolin. Clin J Chin Med. 2022;14:141–5.

[34] Fei J Liang B Jiang C Ni H Wang L. Luteolin inhibits IL-1β-induced inflammation in rat chondrocytes and attenuates osteoarthritis progression in a rat model. Biomed Pharmacother. 2019;109:1586–92. 30551412 10.1016/j.biopha.2018.09.161

[35] Nabavi SF Braidy N Gortzi O. Luteolin as an anti-inflammatory and neuroprotective agent: a brief review. Brain Res Bull. 2015;119:1–11. 26361743 10.1016/j.brainresbull.2015.09.002

[36] Nguyen TLA Bhattacharya D. Antimicrobial activity of Quercetin: an approach to its mechanistic principle. Molecules. 2022;27:2494. 35458691 10.3390/molecules27082494 PMC9029217

[37] Wu W Li R Li X. Quercetin as an antiviral agent inhibits influenza a virus (IAV) entry. Viruses. 2016;8:6. 10.3390/v8010006 PMC4728566 26712783

[38] Tang J Diao P Shu X Li L Xiong L. Quercetin and Quercitrin attenuates the inflammatory response and oxidative stress in LPS-induced RAW264.7 cells: in vitro assessment and a theoretical model. Biomed Res Int. 2019;2019:7039802. 31781635 10.1155/2019/7039802 PMC6855062

[39] Qi W Qi W Xiong D Long M. Quercetin: its antioxidant mechanism, antibacterial properties and potential application in prevention and control of toxipathy. Molecules. 2022;27:6545. 36235082 10.3390/molecules27196545 PMC9571766

[40] Hong Z Meng L Ge Y Zhang Y. In vitro bateriostasis of Qucercetin against Escherichia coli and staphlococcus aureus. J Jilin Inst Chem Technol. 2017;34:38–41.

[41] Zhang R Li G Zhang Q. Hirsutine induces mPTP-dependent apoptosis through ROCK1/PTEN/PI3K/GSK3β pathway in human lung cancer cells. Cell Death Dis. 2018;9:598. 29789524 10.1038/s41419-018-0641-7 PMC5964100

[42] Wang C Li X Zhang J Yao K Hua L Hu J. Anti-tumor effect of hirsutine regulated Shh signaling pathway on colorectal cancer-bearing mice and effect of CD4+ and CD8+ T cells. Chin J Immunol. 2022;38:2473–8.

